# Immune cell metabolic dysfunction in Parkinson’s disease

**DOI:** 10.1186/s13024-025-00827-y

**Published:** 2025-03-24

**Authors:** Julian R. Mark, Malú Gámez Tansey

**Affiliations:** 1 Department of Neuroscience, College of Medicine, University of Florida, Gainesville, FL 32610 USA; 2 Center for Translational Research in Neurodegenerative Disease, College of Medicine, University of Florida, Gainesville, FL 32610 USA; 3 McKnight Brain Institute, University of Florida, Gainesville, FL 32610 USA; 4 Department of Neurology and Norman Fixel Institute for Neurological Diseases, University of Florida Health, Gainesville, FL 32608 USA

## Abstract

Parkinson’s disease (PD) is a multi-system disorder characterized histopathologically by degeneration of dopaminergic neurons in the *substantia nigra pars compacta*. While the etiology of PD remains multifactorial and complex, growing evidence suggests that cellular metabolic dysfunction is a critical driver of neuronal death. Defects in cellular metabolism related to energy production, oxidative stress, metabolic organelle health, and protein homeostasis have been reported in both neurons and immune cells in PD. We propose that these factors act synergistically in immune cells to drive aberrant inflammation in both the CNS and the periphery in PD, contributing to a hostile inflammatory environment which renders certain subsets of neurons vulnerable to degeneration. This review highlights the overlap between established neuronal metabolic deficits in PD with emerging findings in central and peripheral immune cells. By discussing the rapidly expanding literature on immunometabolic dysfunction in PD, we aim to draw attention to potential biomarkers and facilitate future development of immunomodulatory strategies to prevent or delay the progression of PD.

## Introduction

Parkinson’s disease (PD) is the second most common neurodegenerative disorder after Alzheimer’s disease (AD), affecting millions worldwide [1]. The cardinal motor symptoms of PD, which include tremor, rigidity, akinesia, and postural instability result from degeneration of dopaminergic neurons (DANs) within the *substantia nigra pars compacta* (*SNpc*) [2]. The exact cause of neuronal death in PD is not fully understood; however, growing evidence points to bioenergetic insufficiency within DANs as a penultimate step prior to degeneration [3–5]. Indeed, many of the known contributors to PD such as normal aging, genetic predisposition, and exposure to environmental toxins converge on metabolic dysfunction [6, 7]. Importantly, recent evidence suggests that many defects in energy homeostasis reported in PD DANs, including poor mitochondrial and lysosomal health, are also observed in immune cells [8–10]. Therefore, immune cell metabolic dysfunction in PD may have broad scientific and clinical implications ranging from mechanistic understanding of disease progression to biomarkers for improving patient care. In this review, we will outline the current understanding of immune cell metabolic dysfunction in PD including changes in glycolytic activity, mitochondrial and lysosomal deficits, and disrupted homeostasis of fundamental energetic substrates. We will highlight areas of overlap between the deficits reported in immune cells and those previously reported in neurons, and we will identify gaps in the literature which future research should seek to address.

## Glycolysis

Glycolysis is one of the most fundamental pathways of energy production for mammalian cells [11], and glucose utilization is known to be disrupted in the brains of individuals with idiopathic PD (iPD) compared to age-matched non-PD controls [12]. Glycolysis is a highly conserved process across many domains of life that occurs in the cytoplasm, and it consists of a series of reactions that extract energy from glucose [13]. This requires an energy investment phase which consumes ATP to activate glucose, then an energy payoff phase that generates ATP and NADH through substrate-level phosphorylation and redox reactions [13]. The end product of glycolysis is pyruvate, which can be oxidized to Acetyl-CoA and used to generate electron carriers such as NADH and FADH_2_ in the Krebs cycle [14]. These energy-rich molecules are used in oxidative phosphorylation (OXPHOS) at the inner mitochondrial membrane to generate large amounts of ATP; in contrast to glycolysis alone which produces a net of 2 ATP per glucose, OXPHOS in combination with glycolysis increases the yield to 32–34 ATP per glucose molecule [15]. We direct the reader to other excellent reviews for more detailed explanations on these processes [13, 16, 17]. Growing evidence suggests that neurodegenerative diseases, including PD, are linked to metabolic reprogramming in immune cells that shifts the balance in cellular decision-making between glycolysis and OXPHOS [18, 19]. This could potentially contribute to bioenergetic deficits in PD that prime immune cells to respond with hyperinflammatory responses to stimulation [20].

**Fig. 1:**
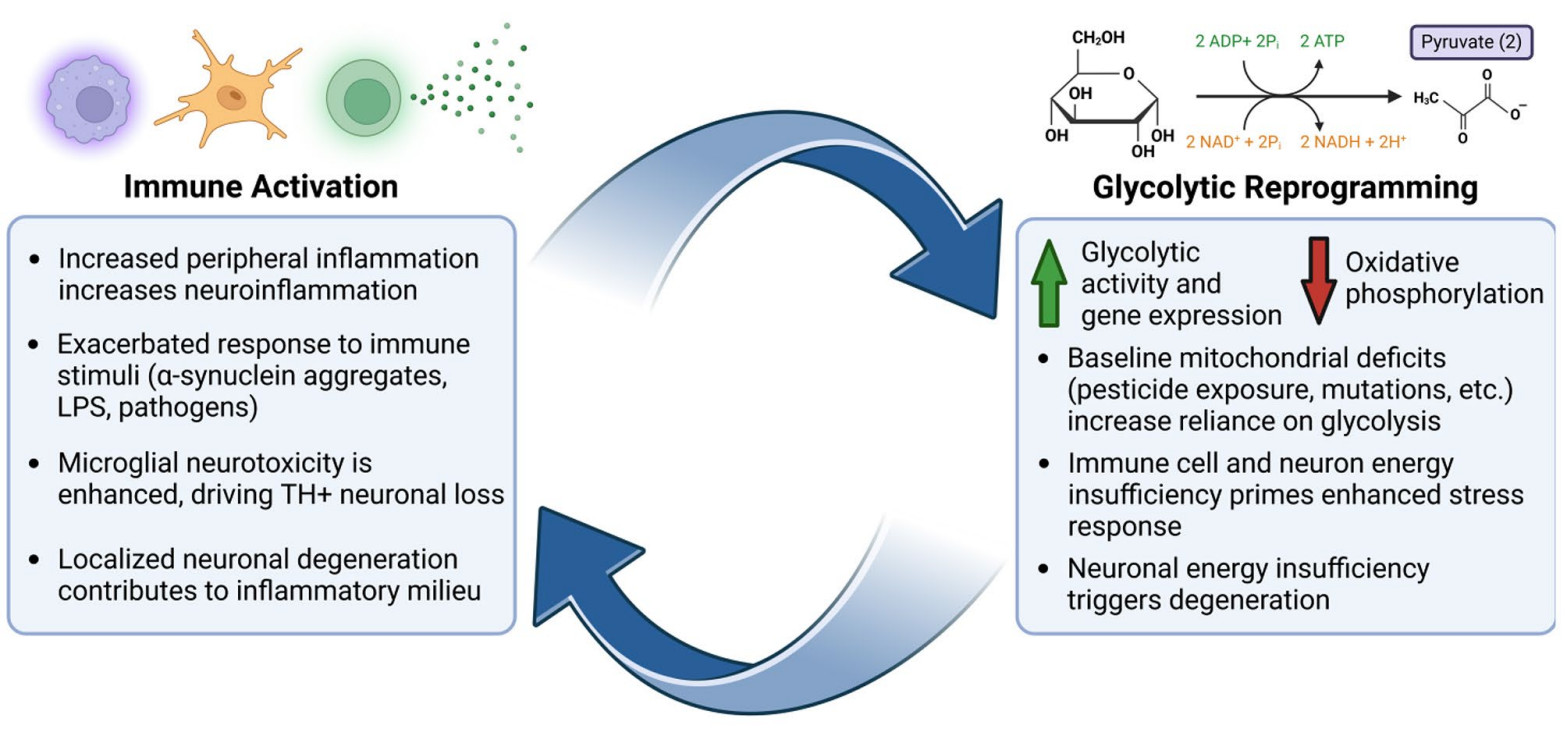
Proposed bidirectional and cyclical relationship between altered immune activation and glycolytic reprogramming in PD. This figure presents a schematic overview of factors which contribute to dysregulated immune function and increased glycolytic activity in the context of PD. Exposure to immune stimuli causes pro-glycolytic reprogramming in both central and peripheral immune cell subtypes. Interventions and underlying deficits which increase cellular utilization of glycolytic pathways have been shown to prime enhanced pro-inflammatory responses to secondary insults. These elements combine to create a feed-forwards cycle that contribute to dopaminergic neuron loss in PD. Abbreviations: lipopolysaccharide (LPS); tyrosine hydroxylase (TH). *Created with BioRender.com*

A wealth of evidence suggests that glycolytic pathways are dysregulated in preclinical models of PD. Induced pluripotent stem cell (iPSC)-derived neurons harboring PD-associated mutations display marked dysregulation in glycolytic gene expression and reduced levels of glucose, ATP, and pyruvate [21]. In addition, treatment with 1-methyl-4-phenyl-1,2,3,6-tetrahydropyridine (MPTP), a compound frequently used to model PD-like degeneration in animals, causes reductions in glycolytic activity, ATP, and pyruvate in human neuroblastoma cells [22]. Dysregulated glycolysis has also been observed in immune cells in various preclinical PD models (Fig. 1). Mouse microglia treated with α-synuclein preformed fibrils (PFFs) display increased glycolytic activity and capacity with simultaneous reductions in mitochondrial basal respiration and ATP production [23]. Furthermore, inhibition of glycolytic activity has been shown to ameliorate microglial activation and TH^+^ neuronal loss in both lipopolysaccharide (LPS)- and MPTP-induced mouse models of PD-like nigrostriatal degeneration [24]. These findings suggest that increased glycolysis in microglia is associated with a pro-inflammatory state, potentially exacerbating the vulnerability of nearby neurons and contributing to eventual neuronal degeneration in PD [20]. Supporting this, reports indicate that primary microglia from mice with heterozygous knockout (KO) of *Clk1*, a gene encoding a mitochondrial hydroxylase, exhibit elevated production of proinflammatory cytokines and reactive oxygen species (ROS) [25]. Consistent with this finding, *Clk1*^−/−^ BV2 microglia display a hyperinflammatory phenotype and a shift towards aerobic glycolysis that is abrogated by pharmacologic glycolysis inhibitors [25]. Although not of immune cell origin, recent evidence suggests that in PD, astrocytes which are capable of mounting inflammatory responses [26, 27], may show similar pro-glycolytic reprogramming. Specifically, astrocytes derived from PD patients carrying mutations in leucine-rich repeat kinase 2 (*LRRK2*) display significantly increased basal and compensatory glycolytic activity compared to controls [28], and *LRRK2* PD astrocytes produce higher levels of IL-6 than control astrocytes after exposure to tumor necrosis factor (TNF) and interleukin-1 beta (IL-1β) [29]. In summary, these studies suggest a role for CNS metabolic deficits in immune and astroglia activation within the context of PD neurodegeneration, making it a promising target for future clinical development.

Similar findings have been reported in the peripheral immune system, with peripheral blood mononuclear cells (PBMCs) from individuals with iPD exhibiting increased glycolytic activity, capacity, and reserve relative to controls [30]. Intriguingly, these same glycolytic changes are observed in PBMCs from those with rapid eye movement (REM) sleep behavior disorder (RBD), a group generally considered to approximate prodromal PD due to their high likelihood of converting to PD or related synucleinopathies [31]. RBD is characterized by loss of muscle atonia during REM sleep and the physical acting out of dreams that are often intense or violent [32]. Approximately 80% of individuals with RBD will develop a neurodegenerative disease within 10.5 years of RBD diagnosis, and the plurality (43%) of those who convert will develop PD [31]. Therefore, the finding that peripheral immune cell glycolytic activity is dysregulated prior to the motor stage of PD has significant implications for understanding disease progression. Increased glycolytic activity in PBMCs may contribute to a hyperinflammatory response in the periphery, mirroring the findings in microglia [24, 25] (Fig. 1). Indeed, plasma levels of the cytokine TNF are increased in RBD patients [33], and early-stage PD patients show elevated serum levels of IL-1β^34^. Moreover, pharmacologic inhibition of glycolysis was shown to attenuate inflammatory responses in several mouse models of peripheral inflammation including irritable bowel disease, rheumatoid arthritis, and acute respiratory distress syndrome [35]. In sum, these results suggest that glycolytic activity is increased in both central and peripheral immune cells in individuals with non-motor features of PD, and that targeting glycolysis may be an effective strategy to mitigate aberrant inflammation in the prodromal stage of PD.

## Mitochondrial dysfunction

Mitochondria are rod-shaped organelles surrounded by a double membrane and are colloquially known as the “powerhouse of the cell” due to their crucial role in generating energy through OXPHOS and the electron transport chain [17]. As mentioned above, OXPHOS occurs at the inner mitochondrial membrane and constitutes the largest source of energy production in eukaryotic cells [36]. This process involves a series of redox reactions where electrons are transferred from high-energy carriers including NADH and FADH_2_ through protein complexes (I-IV) and mobile carriers like ubiquinone and cytochrome c [17, 37]. As electrons move through the electron transport chain (ETC), the energy released at each step is used to actively pump protons into the intermembrane space and generate an electrochemical gradient [17]. Protons are allowed to flow back into the matrix through a molecular turbine known as ATP synthase (complex V), which harnesses the proton motive force to phosphorylate ADP to ATP [37]. The final step of the ETC involves molecular oxygen acting as the terminal electron acceptor [37]; this represents a crucial difference from glycolysis which can occur in the absence of oxygen [13, 17]. For additional details on mitochondrial function and dysfunction, we direct the reader to comprehensive reviews on mitochondrial energy production [17, 37].

**Fig. 2:**
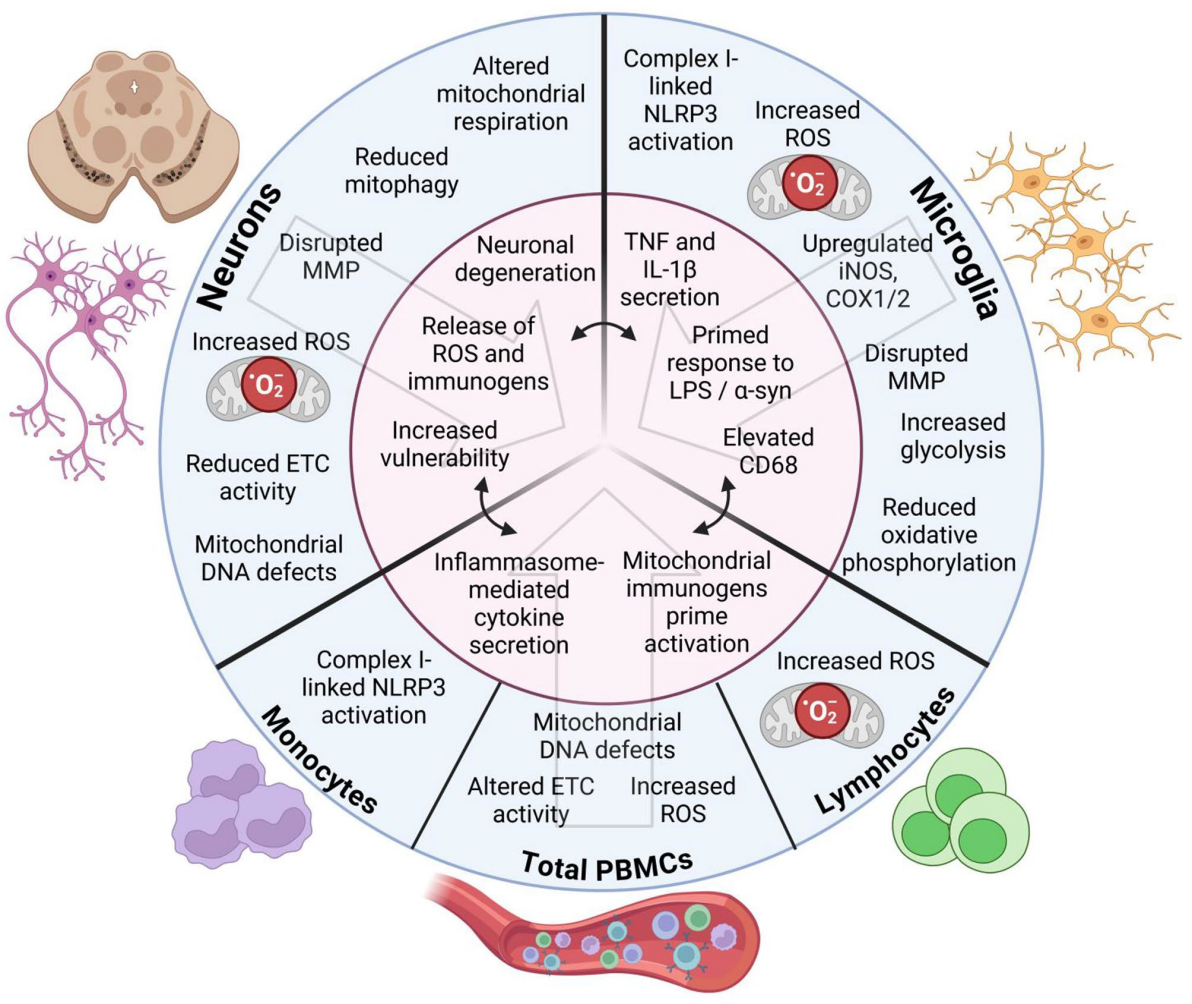
Known facets of mitochondrial dysfunction in PD patients and preclinical models which converge on immune dysregulation. Widespread mitochondrial dysfunction is established to occur within neurons in the etiology of PD, including deficits in electron transport chain activity, respiration, ROS homeostasis, and mitochondrial membrane potential. Several of these deficits have been reflected in immune cells derived from PD patients and preclinical models of PD-like degeneration. Mitochondrial dysfunction can increase inflammasome-mediated cytokine secretion and pro-inflammatory response to aggregated α-synuclein. Inflammation promotes increased central-peripheral immune crosstalk and the formation of an inflammatory milieu that is hostile to vulnerable neurons. ROS from dopamine metabolism may render dopaminergic neurons particularly susceptible to degeneration, and a “second hit” of mitochondrial dysfunction overwhelms their capability to survive an inflammatory, neurotoxic environment. Abbreviations: mitochondrial membrane potential (MMP); reactive oxygen species (ROS); electron transport chain (ETC); inducible nitric oxide synthase (iNOS); cyclooxygenase (COX); tumor necrosis factor (TNF); lipopolysaccharide (LPS). *Created with BioRender.com.*

Genetic findings have extensively implicated mitochondrial dysfunction in PD pathogenesis. For example, Parkin, an E3 ubiquitin ligase encoded by the *PRKN* gene, ubiquitinates damaged mitochondria to induce degradation and mitophagy [38], and mutations in Parkin are the most common cause of autosomal recessive early-onset PD [38]. Parkin deficiency compromises mitochondrial function in *Drosophila* [39], mice [40], and iPSC-derived neurons from individuals with *Parkin* PD [41]. Further strengthening the connection between mitochondria and PD, pathogenic variants in the Parkin regulatory protein PTEN-induced kinase 1 (PINK1) form the second most common cause of autosomal recessive PD [42]. PINK1 recruits Parkin to damaged mitochondria and phosphorylates Parkin along with its ubiquitin conjugates on the outer mitochondrial membrane to regulate autophagic clearance of damaged mitochondria [43]. Sporadic forms of PD have also been linked to deficits in mitochondrial health, with iPD neurons showing decreased expression of mitochondrial complexes I–V [44] (Fig. 2). In addition, fibroblasts from individuals with iPD display decreased mitochondrial connectivity, reduced branching, decreased mean mitochondrial size, and increased fragmentation [45]. Mitochondrial deficits in non-immune cells have been extensively reviewed by others, and we refer the reader to these articles for a more comprehensive breakdown of the subject [46, 47] in addition to detailed reviews discussing the known genetic risk factors for PD [48, 49]. Here, we focus on the growing evidence implicating mitochondrial dysfunction in immune cells as a driver of PD pathogenesis.

### Electron transport chain defects

The ETC is an essential component of effective mitochondrial function, and growing evidence suggests that disrupted ETC function is linked to PD pathogenesis. For instance, *SNpc* samples from individuals with iPD show significantly decreased activity of mitochondrial complex I relative to controls [50], while PD astrocytes exhibit reduced expression of complexes I-V [51]. While the effects of reduced ETC activity and energy production may directly compromise neuronal survival, recent findings also suggest that ETC dysfunction in microglia may contribute to PD through increasing neuroinflammation (Fig. 2). Work by Sarkar et al. demonstrated that LPS-primed primary mouse microglia significantly upregulated IL-1β secretion in a dose-dependent manner after treatment with the mitochondrial complex I inhibitors rotenone and tebufenpyrad [52]. Rotenone is a pesticide epidemiologically linked to increased PD risk [53, 54]; however it did not increase inflammatory cytokine secretion in unprimed microglia in this study [52]. This suggests that inhibition of mitochondrial respiration is not sufficient to drive inflammation but may potentiate the proinflammatory response to a secondary insult. Importantly, secretion of IL-1β, but not TNF, was significantly affected by rotenone treatment, indicating that complex I inhibition in primed microglia likely acts through inflammasome signaling. To confirm this, pre-treatment with an NLRP3 inflammasome inhibitor completely blocked the rotenone-induced increase in IL-1β secretion but did not alter secretion of IL-12, as IL-12 processing is independent of NLRP3 activation [52]. Similar findings were reported by Won et al. who demonstrated that rotenone treatment of mouse bone marrow-derived macrophages (BMDMs) primed NLRP3 inflammasome activation [55]. Importantly, co-treatment with ATP and rotenone resulted in NLRP3-dependent caspase-1 activation whereas rotenone treatment alone had no significant effect [55]. Overall, these results point to a mechanism whereby baseline reductions in mitochondrial complex I activity in PD contribute to excessive inflammation through upregulation of inflammasome activity.

In a recent study, peripheral blood lymphocytes from individuals with iPD were found to have diminished mitochondrial complex II activity relative to age-matched controls [56]. The activity of complexes I, III, and IV was similar across PD lymphocytes and controls; however, activity of complex I was significantly decreased in PD platelets [56]. On the other hand, Müftüoglu et al. observed reduced activity of complexes I and IV in iPD PBMCs, in addition to decreased complex I activity in *Parkin* PD relative to controls [57]. Discrepancies between these studies may arise from the use of PBMCs versus specifically lymphocytes. Therefore, further study is necessary to develop a consensus on which ETC complexes are disrupted in peripheral immune cells in PD and to what extent. Interestingly, iPD PBMCs display a significant upregulation in mRNA expression of the respiratory chain subunits that compose complexes I–V [58]. This may represent a compensatory response to ETC deficits in these cells, suggesting that strategies to rescue ETC activity could have therapeutic potential for restoring peripheral immune function in PD.

### Changes in mitochondrial respiration

Deficits in mitochondrial respiration and oxygen consumption have been a focus of PD research since the discovery that inhibition of mitochondrial complex I causes parkinsonian symptoms in humans and animal models [59, 60]. Basal respiration and passive proton leak across the mitochondrial membrane are increased in iPSC-derived neural cells from *PINK1* PD patients, while neural cells from those with *LRRK2* G2019S PD show significantly lower basal respiration relative to controls [61]. Meanwhile, *PINK1* KO and chronic MPTP treatment in aged mice have been shown to significantly reduce the maximal respiratory rate of mitochondria from the striatum [62, 63].

More recent studies have sought to determine if PD is associated with respiratory defects in CNS immune cells, given the possibility that energy insufficiency could contribute to inflammatory changes. Lu et al. reported that microglia treated with α-synuclein PFFs underwent significant metabolic reprogramming, demonstrating a reduction in basal and maximal respiratory capacity measured by oxygen consumption rate (OCR) [23]. Concurrently, PFF treatment caused a decrease in microglial ATP production and an increase in extracellular acidification rate (ECAR), indicating a shift from OXPHOS towards energy-inefficient glycolysis. These findings indicate that exposure to immunogenic stimuli such as misfolded α-synuclein can drive reductions in mitochondrial respiration in microglia. Moreover, impaired OXPHOS in microglia is accompanied by increased microglial reactivity, with PFF treatment also causing increased expression of TNF and IL-1β^23^ (Fig. 2). Microglial metabolic deficits were rescued by pre-treatment with capsaicin [23], a TRPV1 agonist which has previously been shown to inhibit oxidative stress and attenuate DAN degeneration in MPTP-treated mice [64]. These findings were supported by another group who reported that inhibition of mitochondrial complex V in IL-4 treated microglia caused a downregulation of anti-inflammatory markers, leading to an overall more pro-inflammatory phenotype [65]. Sonninen et al. reported that iPSC-derived astrocytes from individuals with *LRRK2* PD displayed decreased maximal and spare respiration [29], reflecting a similar pattern of metabolic changes observed by Lu et al. involving α-synuclein PFF treated microglia [23]. Jointly, these reports suggest that preventing maladaptive metabolic reprogramming of inflammatory cells in the CNS and preserving mitochondrial respiration may help to combat neuroinflammation in PD.

Defects in mitochondrial respiration in some peripheral cell types like fibroblasts [66, 67] have been documented in PD, but a consensus has yet to develop on whether this extends to peripheral immune cells. Smith et al. reported that PBMCs from individuals with RBD and iPD showed no significant differences in basal respiration, maximal respiration, spare capacity, ATP production, and nonmitochondrial respiration relative to controls [30]. In contrast, Schirinzi et al. observed increased maximal and spare respiratory capacity in iPD PBMCs compared to neurologically healthy controls (NHCs) [68]. Furthermore, maximal and spare respiratory capacity in PD PBMCs were significantly positively correlated with disease duration and degree of motor impairment as measured by MDS-UPDRS and Hoehn & Yahr scores [69]. It is possible that PBMCs adaptively increase their total mitochondrial content over the course of disease as a compensatory response to intermittent spikes in energetic demand. A separate study by Annesley et al. evaluated immortalized lymphoblastoid cell lines derived from iPD PBMCs and reported elevated basal mitochondrial activity and ATP synthesis [70]. This pattern of mitochondrial dysfunction differs from that observed by Schirinzi and colleagues, and instead suggests a higher baseline energetic demand in PD. The variability observed across reports could be attributed to multiple factors including insufficient power and the possibility that different subsets of PBMCs are differentially affected by PD status in terms of respiratory defects. For example, lymphocytes have been shown to exhibit a higher OCR/ECAR ratio than monocytes, indicating a greater utilization of oxidative phosphorylation in lymphocytes [71]. Therefore, future studies should address the possibility of underlying differences in PBMC population frequencies to reduce this potential source of variability.

### Oxidative stress and mitochondrial reactive oxygen species

The mitochondrial ETC is the major source of intracellular ROS production [72], and mitochondrial dysfunction can lead to increased levels of superoxides, hydrogen peroxide, and hydroxyl radicals [73]. These ROS can cause significant damage to the cell by oxidizing proteins, DNA, and lipids, thereby compromising vital cell functions and viability [74]. Recent studies have revealed increased ROS levels [75], reduced function of antioxidant peroxidases [76], and decreased levels of glutathione, the primary endogenous antioxidant molecule, in the *SNpc* of PD patients relative to age-matched controls [77] (Fig. 2). Furthermore, PD-associated mutations in *LRRK2*, *ATP13A2*, *DJ-1*, *SNCA*, *Parkin*, and *PINK1* are associated with increased levels of ROS in cellular and animal models [78–85]. While excessive ROS is known to be directly neurotoxic, there is a growing recognition for the role of ROS in immune activation. Here, we will discuss the evidence supporting dysregulated ROS homeostasis in immune cells in PD, as well as potential mechanisms connecting oxidative stress to immune-mediated neurodegeneration.

Markers of elevated oxidative stress have been described in CNS immune cells from individuals with PD and animal models of disease. Hunot et al. reported that microglia in the *SNpc* and ventral tegmental area (VTA) of individuals with PD express increased levels of inducible nitric oxide synthase (iNOS) [86]. Knott et al. replicated these findings and observed that *SNpc* microglia from PD patients but not controls displayed upregulation of iNOS, cyclo-oxygenase (COX)-1, and COX-2^87^. In mouse models of PD-like degeneration, administration of LPS and nitrated α-synuclein led to increased ROS production in microglia [88–90]. Furthermore, mice with microglial-specific overexpression of α-synuclein showed upregulation of *SOD1* and *SOD2* in microglia, indicating a disrupted oxidative status [90]. Microglial ROS production in response to α-synuclein can be attenuated through knockout of NADPH oxidase [88], an enzyme whose function is to catalyze the production of superoxide and hydrogen peroxide by transferring electrons to molecular oxygen. Interestingly, α-synuclein-induced ROS production was also attenuated by blockade of voltage-gated potassium and chloride currents in microglia [91], suggesting that excitotoxicity can exacerbate microglial oxidative stress. Limited evidence suggests that PD is also associated with disrupted ROS homeostasis in astrocytes, with one group reporting that iPSC-derived astrocytes from PD patients express increased levels of oxidized proteins compared to controls [28].

Recent findings suggest that drivers of oxidative stress in microglia lead to downstream immune activation, creating a cytotoxic milieu that drives DAN degeneration in PD. Mouse microglia overexpressing human α-synuclein exhibit increased ROS and expression of CD68, a marker of activated microglia, compared to wild type cells [90]. Accumulation of α-synuclein in microglia was also accompanied by upregulation of proinflammatory chemokines and infiltration of T cells into the *SNpc* [90]. Furthermore, chronic rotenone treatment of microglia in vitro leads to increased ROS production and microglial activation [92, 93], consistent with prior research showing that mitochondrial ROS directly activate the NLRP3 inflammasome [94, 95]. Administration of iNOS inhibitors in mice with microglial-specific overexpression of human α-synuclein mitigated ROS generation, microglial activation, and TH^+^ DAN loss [90]. These findings highlight microglial ROS production as a relevant therapeutic target for future studies. Overall, these results suggest that oxidative stress in microglia is a critical component of the pathogenic cascade, upstream of immune activation and DAN degeneration in PD.

Studies thus far suggest that disrupted ROS homeostasis is not limited to CNS immune cells but may be present in peripheral blood immune cells. Prigione et al. described increased intracellular ROS in PD PBMCs compared to matched controls, with no change in glutathione reductase activity [96]. The authors also observed that daily levodopa dosage in the PD group was inversely correlated with ROS production, suggesting that levodopa may play a protective role in the redox status of PBMCs [96]. Similarly, lymphoblastoid cell lines derived from individuals with iPD displayed elevated ROS production compared to those derived from controls [70]. On the other hand, Smith et al. observed that PD monocytes displayed increased absolute ROS levels and ROS normalized to mitochondrial content, but PBMCs as a whole did not show significant differences [30]. ROS homeostasis is particularly relevant to monocytes due to their employment of ROS as a bactericidal agent against engulfed pathogens [97]. It will be vital to determine whether ROS homeostasis in PD shows cell-type specific differences, as focusing on the most disease-relevant cell types could enhance the sensitivity of future studies to detect immunometabolic deficits.

### Disruption in mitochondrial membrane potential

Mitochondrial health can be measured in many ways, but one of the most common metrics is mitochondrial membrane potential (MMP). The maintenance of an electric potential across the inner mitochondrial membrane (negatively charged in the matrix) is necessary to power ATP synthase, and sustained decreases in MMP trigger mitophagy and overall decreases in cell viability [98]. A number of PD-related genes are known to maintain MMP in neurons. For example, knockdown (KD) of *PINK1* in mouse DANs has been shown to cause disrupted calcium homeostasis, increased ROS production, and a reduced MMP [99]. Similarly, loss of *DJ-1* causes reduced membrane potential and increased mitochondrial fragmentation [100].

Emerging literature suggests that immune cell mitochondrial health is compromised in PD models. For example, iPSC-derived astrocytes from individuals with *LRRK2* PD have reduced MMP and increased mitochondrial fragmentation relative to controls [28]. Experimentally, chronic treatment with α-synuclein PFFs can reduce microglial MMP [23], indicating that aggregated α-synuclein may compromise mitochondrial membrane integrity. Indeed, Choi et al. reported that the mitochondrial lipid cardiolipin can catalyze the oligomerization of A53T α-synuclein, and the buildup of α-synuclein aggregates at mitochondria causes permeabilization of mitochondrial membranes and cell death [101]. Mouse models of *PARK7*/DJ-1 KO show no differences in microglial MMP at baseline, however LPS treatment significantly reduces microglial MMP in DJ-1 KO relative to wild type [102]. These results imply that loss of DJ-1 impairs mitophagy and renders microglia vulnerable to other stresses, which is consistent with previous reports in neurons describing DJ-1 as a mediator of PINK1/Parkin-dependent mitophagy [103]. In the periphery, Smith et al. reported that iPD PBMCs do not exhibit alterations in MMP relative to controls [30], in contrast to Qadri et al. who observed significantly reduced MMP in PD PBMCs relative to controls in both stimulated and unstimulated conditions [104]. This discrepancy could be due to the different probes used by each group; Smith et al. used tetramethylrhodamine methyl ester, while Qadri et al. used JC-1^30,104^. The possibility of using peripheral immune cell MMP as a biomarker for PD is exciting, however it will be vital for the field to develop a consistent methodology for evaluating MMP before these findings can be translated into providing clinical benefit.

### Mitophagy defects

Mitophagy is an essential quality-control process wherein damaged mitochondria are targeted, engulfed by autophagosomes, and ultimately degraded by lysosomes [105]. Defects in mitophagy have been heavily implicated in PD [103, 106–108], and mitophagy in immune cells has received attention due to studies linking the accumulation of damaged mitochondria to inflammasome activation [109]. Therefore, it is possible that disease-modifying therapies aimed at enhancing mitophagy in PD will both improve neuronal viability and combat aberrant immune activation. In a study by Singh et al., mice carrying the pathogenic *LRRK2* G2019S mutation showed reduced basal mitophagy in both neurons and microglia, while *LRRK2* KO was associated with increased mitophagy [107]. Furthermore, pharmacologic inhibition of LRRK2 kinase activity rescued these mitophagy defects in vivo [107]. It has also been shown that mice with a microglia-specific knockout of autophagy protein-5 (*Atg5*), a protein known to mediate mitophagy [110], exhibit poorer performance in Rotarod and Morris water-maze tasks as well as loss of TH^+^ neurons^111^. Microglia with *Atg5* KO showed increased activation of the NLRP3 inflammasome, and pharmacologic inhibition of inflammasome activity caused reduced immune activation and reduced TH^+^ neuronal loss [111]. Studies using LPS and MPTP mouse models have reported similar findings, with pharmacologic activators of mitophagy causing reduced microglial activation and improved DAN viability [112, 113]. Taken together, these findings provide strong evidence that mitophagy deficits specifically in microglia can contribute to PD-like degeneration, and this is likely driven by inflammasome-mediated immune activation.

### Mitochondrial DNA defects

Mitochondria are unique among cellular organelles as they contain their own DNA, and the cellular mechanisms for replicating and proofreading mitochondrial DNA (mtDNA) are distinct from those used for nuclear DNA [114]. Postmortem samples of the *SNpc* from PD donors show an increased number of mtDNA deletions and rearrangements relative to NHCs and AD, suggesting that these classes of mtDNA defects may be specific to PD pathogenesis [115–117]. Notably, mtDNA defects in PBMCs may have clinical value as a blood-based biomarker for PD. Qi et al. developed a PCR-based assay based on the principle that lesions in mtDNA block the ability of DNA polymerase to replicate, allowing for quantification of mtDNA damage [118]. Using this assay, the authors determined that iPD and *LRRK2* PD PBMCs display significantly increased levels of mtDNA defects, and pharmacologic inhibition of LRRK2 kinase activity effectively reduced the level of mtDNA defects in iPD PBMCs to the same levels observed in NHCs [118]. This promising technique could be leveraged to identify PD endophenotypes using the accessibility of PBMCs, with the potential to improve patient classification into clinical trials for therapeutics aimed at rescuing mitochondrial function. Future efforts should be directed towards investigating if mtDNA defects occur prior to the manifestation of motor symptoms in prodromal PD, and additionally to explore the mechanism by which LRRK2 kinase activity contributes to mtDNA damage.

Defects in mtDNA have also been shown to play a role in immune activation and inflammation. When mitochondria are damaged, mtDNA can leak into the cytoplasm due to mitochondrial outer membrane permeabilization or defective mitophagy [119]. The cytosolic DNA sensor cyclic GMP-AMP synthase (cGAS) recognizes this leaked mtDNA as a danger-associated molecular pattern (DAMP) and catalyzes the production of cyclic GMP-AMP (cGAMP) [119, 120]. cGAMP then binds to and activates stimulator of interferon genes (STING) [120], a key adaptor protein located on the endoplasmic reticulum. STING activation ultimately results in the production of type I interferons and pro-inflammatory cytokines [120]. Hinkle et al. recently observed that double stranded DNA breaks in an α-synuclein PFF mouse model of PD triggered dopaminergic degeneration in a STING-dependent manner [121]. Importantly, STING-deficient mice were protected from dopaminergic neuron loss and motor deficits induced by α-synuclein PFFs [121]. In addition, Hancock-Cerutti et al. reported that KO of *VPS13C*, a gene associated with autosomal recessive PD, also leads to inflammation mediated by mtDNA and the cGAS-STING pathway [122]. The VPS13 gene family is involved in lipid transport to mitochondria and lysosomes [123], and VPS13^KO^ HeLa cells show increased cytosolic mtDNA levels and simultaneous cGAS-STING activation [122]. Depletion of mtDNA with ethidium bromide in these cells was effective in reversing the increased expression of IFN-stimulated genes [122]. These results support a mechanism whereby mtDNA defects and cGAS-STING signaling link mitochondrial dysfunction to immune activation in PD.

## Lysosomal dysfunction and autophagy

Lysosomal dysfunction has emerged as a key player in the pathogenesis of PD. Lysosomes are responsible for the degradation and recycling of intracellular waste, damaged proteins, and cellular debris. One of the hallmark pathological features of PD is the accumulation of abnormal protein aggregates, particularly α-synuclein, within neurons, leading to the formation of Lewy bodies. Growing evidence suggests that impaired lysosomal function plays a key role in the failure to clear these toxic protein aggregates, contributing to the progressive neurodegeneration observed in PD [124, 125]. Some of the most commonly mutated genes in familial cases of PD, including *LRRK2* and *GBA1*, have key functions in regulating lysosomal health [126–128] and both are highly expressed in immune cells [9, 129, 130]. Furthermore, lysosomal and autophagic deficits have been shown to contribute to NF-κB signaling and inflammasome activation [131]. Therefore, emerging literature has begun to identify if immune cells in PD show dysfunction in autophagic flux, vesicle trafficking, and lysosomal degradative capacity.

### Autophagic deficits in PD

Autophagy is the process by which cells break down damaged or unneeded components so that these materials can be repurposed for new cell parts. Autophagic activity protects cellular energy homeostasis by decreasing bioenergetic demand [132], and it plays a vital role in host defense against microbial pathogens following phagocytosis [133]. Neuronal autophagic deficits have been implicated in PD, with *SNpc* samples showing that PD neurons have increased autophagic degeneration including vacuolation of the endoplasmic reticulum and increased lysosome-like vacuoles [134]. In addition, autophagic-lysosomal pathway impairments are known to contribute to the buildup of misfolded proteins including α-synuclein and tau [135, 136]. Furthermore, PD brains display reduced expression of lysosomal-associated membrane protein 2 A (LAMP2A) and heat shock cognate 70 protein (hsc70), which suggests that chaperone-mediated autophagy is reduced in PD [137]. Mutations in several PD-related genes have been linked to autophagic deficits in neurons, including *SNCA*, *LRRK2*, *PARK7/DJ-1*, and *VPS35*^138–141^. Autophagic deficits in PD are also found in immune cells, which is particularly relevant because microglia are the predominant cell type responsible for clearing aggregated α-synuclein in the CNS [142].

Both in vitro and in vivo experiments have demonstrated that α-synuclein exposure suppresses autophagic flux in microglia [90, 143]. Consequently, underlying autophagic deficits may compound upon themselves with the buildup of intracellular α-synuclein aggregates causing further disruption in microglial clearance of misfolded proteins. Nash et al. demonstrated that KD of DJ-1 in microglia significantly decreased phagocytosis of α-synuclein and reduced expression of autophagy-related markers including p62 and LC3-II [144]. DJ-1 KD microglia also showed an enhanced pro-inflammatory response to α-synuclein compared to controls, displaying increased secretion of IL-6, IL-1β, and nitric oxide (NO) [144]. Similarly, autophagic deficits caused by a microglial-specific deletion of *Atg7* in mice caused increased microglial activation [145]. Moreover, microglial-specific KO of *Atg7* exacerbated the pathologic spreading of misfolded tau injected into the brains of mice expressing the P301S mutant form of human tau. Tau is a microtubule associated protein that functions to stabilize microtubules; however, abnormal aggregation of tau has been reported in a variety of neurodegenerative diseases including AD, frontotemporal dementia (FTD), progressive supranuclear palsy, *LRRK2* PD, and others [146, 147]. These results indicate that defective microglial autophagy can drive inflammation in the context of pathologic protein aggregation, thereby promoting DAN degeneration. Indeed, mice with microglial-specific KO of *Atg5* were found to be significantly more vulnerable to MPTP-induced toxicity and exhibited increased DAN degeneration and poorer performance on motor tests compared to WT mice [148]. Mechanistically, it was found that blocking microglial autophagy in vitro with *Atg5* siRNA led to enhanced NLRP3 inflammasome activation after LPS treatment [148].

Several autophagy-related proteins are known to be differentially expressed in peripheral immune cells from individuals with PD. PD PBMCs exhibit increased mRNA and protein expression of autophagy-related p62 relative to matched controls [149, 150]. Additionally, expression of *LAMP2* is significantly decreased in PBMCs from individuals with sporadic PD [151], which may indicate impaired fusion of autophagosomes and lysosomes. A number of other transcripts associated with autophagosome formation (ULK3, ATG2A, ATG4B, ATG5, ATG16L1 and HDAC6) were found to be downregulated in iPD PBMCs, while protein levels of ULK1, Beclin-1, and autophagy/beclin-1 regulator 1 were increased [152]. Expression of these autophagy related proteins positively correlated with increased levels of α-synuclein in PBMCs [152], suggesting that this may represent a compensatory response to trigger increased protein degradation.

The microtubule-associated protein light chain 3 (LC3) is used as a general marker of autophagic activity, with the conversion of cytosolic LC3-I to lipidated LC3-II associated with increased autophagosome formation [153]. In astrocytes, overexpression of WT, A30P, and A53T α-synuclein promoted decreased LC3-II and increased p62 expression, suggesting reduced autophagic activity [154]. Increased LC3 gene expression and LC3-II protein levels has been found in iPD PBMCs [151], and two studies have reported an increased LC3-II/LC3-I ratio [151, 155]. On the other contrary, Miki et al. observed no significant difference in LC3 mRNA or LC3-II/LC3-I ratio in PD PBMCs relative to NHCs [152]. Elevated LC3-II can arise from impaired fusion of autophagosomes with lysosomes [151], therefore it is possible that both LC3-I and LC3-II levels are increased in PD PBMCs due to disruption of downstream clearance of autophagosomes.

### Endolysosomal and vesicle trafficking

#### LRRK2

Mutations in *LRRK2* are the most frequent cause of late-onset autosomal dominant and sporadic PD [156]. Physiologically, LRRK2 is known to participate in a variety of functions related to endolysosomal maturation and trafficking [8, 157]. One of the potential mechanisms by which LRRK2 mutations may contribute to neuronal dysfunction is through impairment of vesicular trafficking. This was established by studies demonstrating that Rab GTPases are bona fide substrates of LRRK2 kinase activity [158–160]. Rab family proteins are known to regulate early and late endocytic trafficking [161], thus changes in their phosphorylation levels caused by gain-of-kinase mutations in LRRK2 may disrupt endosome transport. Additionally, LRRK2 directly interacts with a number of proteins associated with vesicle transport such as actin [162], JIP4^163^, ARFGAP1^164^, VPS52^165^, Sec16a [166], and N-ethylmaleimide sensitive fusion protein (an ATPase that facilitates SNARE complex disassembly) [167].

Similarly, work by our group demonstrated that *G2019S* mutation in murine macrophages caused an increase in phagocytosis and MHC-II trafficking from the perinuclear lysosome to the plasma membrane [168]. We found that macrophages from *G2019S* knock-in mice exhibit an increased ratio of extracellular: intracellular MHC-II which was mitigated by Lrrk2 kinase inhibition. Furthermore, we determined that Lrrk2 modulates antigen presentation through mTOR dependent lysosomal-tubule-formation (LTF). Macrophages with the *G2019S* mutation showed a significant increase in LTF relative to WT, and macrophages nucleofected with *Lrrk2* anti-sense oligonucleotide (ASO) to cause *Lrrk2* KD showed a significant reduction in LTF. Lastly, gene ontology analysis revealed that vesicular trafficking and lysosomal positioning pathways were associated with the response to IFN-γ treatment in *Lrrk2* ASO-nucleofected macrophages. These results highlight that LRRK2 is a key regulator of lysosome and vesicle trafficking, and maintaining proper immune function will be an important consideration for therapeutics which target LRRK2 activity.

#### VPS35

VPS35 is an essential component of the retromer cargo recognition complex which functions in membrane protein transport and trafficking in the trans-Golgi network [169]. Mutations in *VPS35*, especially the D620N mutation, are known causes of familial PD [170, 171], and mouse models carrying *VPS35* D620N have been shown to exhibit Parkinsonian motor features, tau neuropathology, and dopaminergic degeneration [172, 173]. Intriguingly, monocytes and neutrophils derived from individuals with *VPS35* D620N display increased LRRK2-mediated Rab10 phosphorylation compared to controls and iPD, and this difference is abrogated upon LRRK2 kinase inhibition with MLi-2^174^. *VPS35* mutation is not sufficient to cause neuroinflammation, as D620N knock-in mice do not exhibit increased astrogliosis or microgliosis [172, 173], but D620N exacerbates microgliosis secondary to MPTP treatment [172]. Similarly, VPS35 KD in mouse microglia causes an enhanced response to LPS treatment with increased expression of iNOS and IL-6^175^. VPS35 deficiency is also shown to impair Trem2 trafficking back to the trans-golgi network, leading to increased Trem2 accumulation in the lysosome [175], but overexpression of Trem2 mitigates the proinflammatory phenotype in *VPS35* KO microglia [175]. In addition, Pal et al. demonstrated that *VPS35* D620N mouse embryonic fibroblasts show broad changes in lysosomal protein contents and increased recruitment of the phospho-Rab effector protein RILPL1 to the lysosome [176]. *VPS35* D620N lysosomes also showed enhanced recruitment of LRRK2^176^, which may be a potential mechanism to explain the immune priming observed by other groups. Bu et al. reported deficits in dopamine transporter expression in D620N knock-in mice that was rescued by LRRK2 kinase inhibitors [177], but the authors did not investigate immune cell specific effects. Collectively, these results suggest that VPS35 and LRRK2 converge on similar pathways related to immune activation and endolysosomal function. Future effort is warranted to investigate how *VPS35* mutations modulate immune cell function in humans, and whether therapeutics targeting LRRK2 kinase activity may offer clinical benefit to this patient subset.

### Lysosomal degradative capacity

**Fig. 3:**
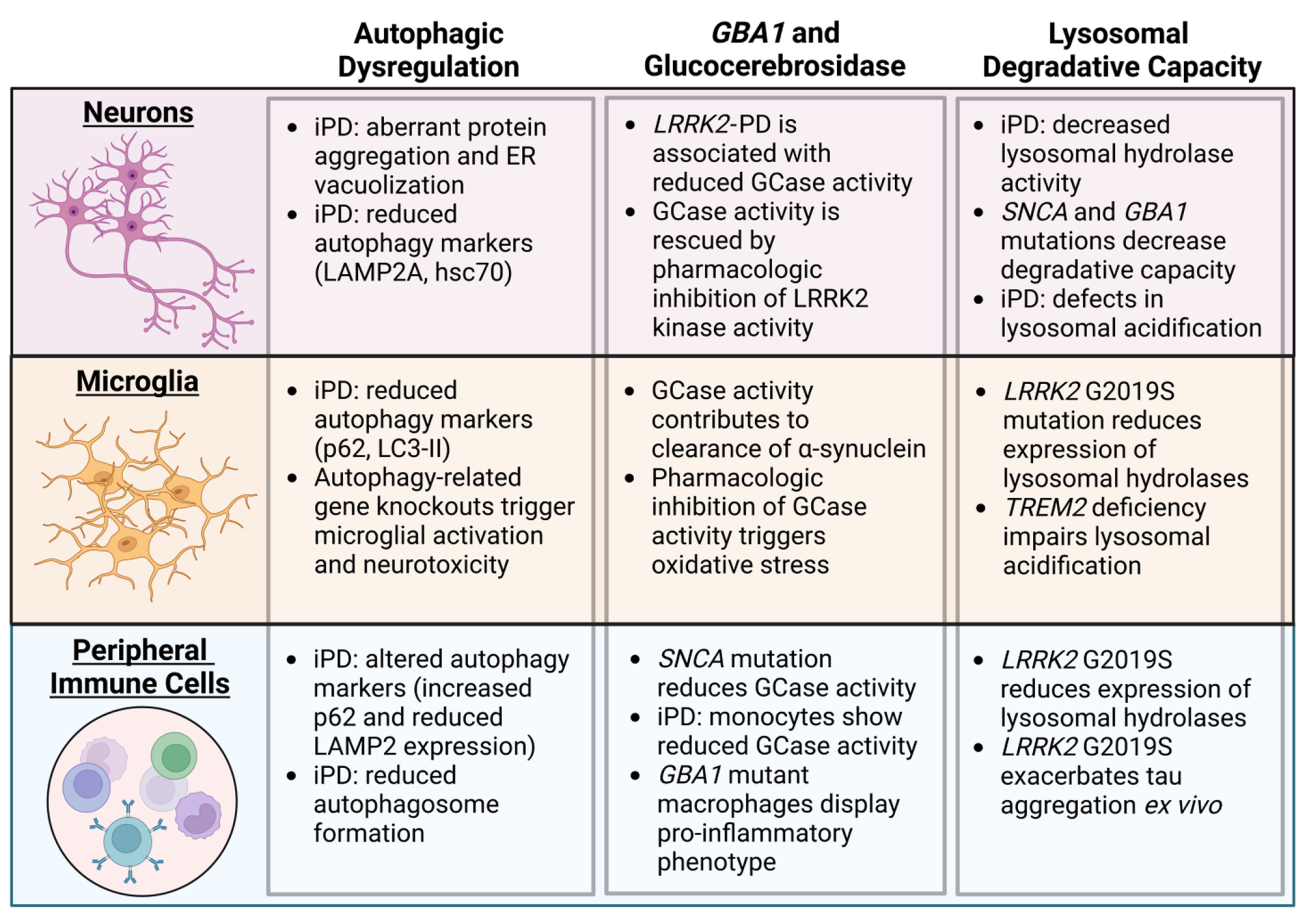
Defects in autophagy and lysosomal function in PD show similarities across neurons and immune cells. This figure highlights areas of autophagic and lysosomal dysfunction that show overlap across neurons, microglia, and peripheral immune cells in PD. These similarities may indicate that peripheral immune cells have potential to be used as an accessible tissue source in clinical practice for the investigation of metabolic deficits in the CNS of PD patients. Therapeutic strategies aimed at rescuing lysosomal function in PD may improve neuronal health while also rescuing lysosomal activity in other cell types. Abbreviations: idiopathic Parkinson’s disease (iPD); endoplasmic reticulum (ER); glucocerebrosidase (GCase); central nervous system (CNS). *Created with BioRender.com*

Decreased activity of lysosomal hydrolase enzymes has been reported in postmortem analyses of the *SNpc* of PD patients [178, 179] and in mouse models of *SNCA* and *GBA1* mutations^180-181^. Multiple lines of evidence now suggest that deficits in lysosomal degradative capacity in PD extend to immune cells in both the CNS and the periphery (Fig. 3). The pathogenic *LRRK2* G2019S mutation is associated with reduced expression of lysosomal hydrolases in macrophages and microglia [182], and astrocytes from *Lrrk2* G2019S mice have reduced cathepsin B activity [183]. Pharmacologic inhibition of LRRK2 kinase activity rescues these phenotypes, indicating that the effects of G2019S on lysosomal degradative capacity are kinase-specific [182, 183]. Human iPSC-derived macrophages with G2019S mutation also show deficits in both internalization and degradation of tau fibrils compared to wild type [184]. Furthermore, non-degraded tau fibrils from *LRRK2* KO macrophages did not retain seeding competency 24 h post incubation, but fibrils from G2019S macrophages retained seeding competency and were able to induce aggregation of naïve tau monomers [184]. These findings suggest that lysosomal degradative activity in immune cells is critical for modulation of toxic protein aggregation in PD.

The vast majority of lysosomal enzymes exhibit optimal function at acidic pH levels [185], thus it is unsurprising that deficits in lysosomal acidification result in decreased degradative capacity [186]. The *SNpc* from individuals with iPD shows significant dysregulation in expression and activity of proteins linked to lysosomal acidification including ATP13A2 and LRRK2 relative to control brains [187, 188]. Intriguingly, *LRRK2* G2019S is associated with reduced lysosomal pH in astrocytes [183] but increased pH in neurons [189], indicating cell-type specific effects. Thus far, it has been shown that increased endogenous expression of α-synuclein disrupts lysosomal pH in iPSC-derived macrophages [190], and impaired lysosomal acidification predisposes microglia to enhanced secretion of proinflammatory cytokines [191]. *TREM2* is a gene involved in lysosomal acidification [192] that has been linked to increased risk for AD [193, 194], with mixed evidence supporting a possible association between *TREM2* variants and increased PD susceptibility [193, 195–197]. TREM2 deficiency was recently shown to cause defective vesicle acidification and heightened immune activation in microglia [192, 198], and KO of Trem2 in mice severely dysregulates neuronal OXPHOS and mitochondrial structure [199]. Together, these results suggest that deficits in microglial lysosomal acidification may contribute to neuroinflammation in PD and may represent a potential therapeutic target. In support of this, pharmacologic treatment to enhance lysosomal acidification was shown to substantially reduce neurodegeneration and microgliosis in a MPTP model of PD-like degeneration [200].

Mutations in the *GBA1* gene which encodes the lysosomal enzyme glucocerebrosidase (GCase) constitute a major genetic risk factor for PD [201, 202], and recent experiments have demonstrated that GCase activity contributes to the clearance of α-synuclein aggregates [203, 204]. These findings have prompted several studies aimed at investigating how GCase activity is altered in microglia and other immune cells in PD. GCase activity has been heavily implicated in microglial activation, with reductions in microglial GCase activity associated with increased neuroinflammation, α-synuclein aggregation, and neurodegeneration [204–207]. Interestingly, genetic ablation of *GBA1* exclusively in midbrain DANs fails to cause degeneration, motor deficits, or α-synuclein aggregation in mice despite significantly increased microglial activation [206]. This suggests that the pathogenic effects of *GBA1* deficiency are likely mediated through non-neuronal cell types. Indeed, mouse astrocytes with knock in of the loss-of-function mutation D409V in *GBA1* show reductions in lysosomal count, increased lysosomal pH, and reduced cathepsin B activity [208]. In addition, Brunialti et al. reported that microglial-specific inhibition of GCase activity with conduritol-B-epoxide in microglia-neuron co-cultures caused a significant reduction in the expression of nuclear factor erythroid 2-related factor 2 (NFE2L2) activity in neurons [209]. NFE2L2 is a transcription factor which regulates the expression of antioxidant proteins and protects against oxidative damage triggered by inflammation; therefore, these findings suggest that deficits in microglial GCase activity can render nearby neurons vulnerable to oxidative stress. These data collectively suggest that microglial GCase activity regulates lysosomal function and is important for protecting neuronal viability in PD.

A number of PD-associated mutations converge on alterations in GCase activity in immune cells (Fig. 3). PBMCs from PD patients carrying *GBA1* and *SNCA* A53T mutations show significant reductions in GCase protein expression relative to NHCs [10]. In contrast, Kedariti et al. observed that *LRRK2* PD PBMCs display increased GCase enzymatic activity relative to both iPD and controls [210]. This implies that LRRK2 kinase activity regulates GCase in a cell-type specific manner, as neurons derived from individuals with *LRRK2* PD show reduced GCase activity which can be rescued by inhibition of LRRK2 kinase activity via MLi-2^211^. Peripheral immune cell subtypes vary in their baseline protein expression of GCase and level of GCase activity [9, 212], and cell-type specific assays may reveal differences that are not detectable in total PBMCs. For example, Atashrazm et al. found that isolated PD monocytes but not lymphocytes displayed reduced GCase activity relative to controls [212]. *GBA1* mutant monocyte-derived macrophages exhibit autophagic defects and increased secretion of IL-1β and IL-6^213^, consistent with the hyperinflammatory phenotype reported in *GBA1* mutant microglia. These defects were attenuated by treatment with the pharmacologic GCase activator NCGC758^213^, indicating that rescue of GCase activity could have therapeutic benefits in preventing aberrant inflammation.

GCase activity in peripheral immune cells may relate to clinical characteristics of PD. Higher GCase activity in blood and peripheral immune cells from individuals with iPD was associated with younger age at onset, longer disease duration, greater levodopa use, and poorer performance on UPDRS and Montreal Cognitive Assessment [214, 215]. This is unexpected given that GCase activity in the blood is modestly lower in those with PD relative to NHCs [214]. The current literature points to GCase activity and lysosomal function as major players in the role of immune cells in PD, however significant gaps in our understanding remain, in particular regarding how different *GBA1* mutations may not produce a uniform phenotype. The L444P variant in *GBA1* is associated with a complete loss of GCase activity [216], whereas the N370S substitution is associated with retention of approximately 10% of the activity of WT [217]. Many of the studies performed thus far have grouped all *GBA1* mutations under a single umbrella due to the difficulty in powering comparisons between genotypes. However, greater consistency in genotyping, reporting, and comparing the effects between *GBA1* mutations in study populations moving forwards will be pivotal to defining the mechanisms linking GCase to PD.

## Ubiquitin-proteasome function

The presence of intracellular inclusions containing aggregated α-synuclein is one of the defining histopathological hallmarks of PD [218], and a wealth of evidence supports the role of dysregulated protein homeostasis in PD pathogenesis. Bioenergetic deficits in neurons have been proposed as a cause of impaired protein quality control in PD [4], and conversely, protein homeostasis is essential for maintaining a number of cellular pathways including autophagy [219] and mitochondrial function [220, 221] which support bioenergetic sufficiency. The ubiquitin-proteasome system (UPS), a major pathway responsible for targeted protein degradation within the cell, has been strongly implicated in the etiology of PD [222, 223]. In this process, proteins are marked for degradation by covalent attachment of ubiquitin molecules and then degraded by a large intracellular protein complex known as a proteasome which releases the reusable ubiquitin [224].

Inhibition of UPS function can compromise cellular metabolism, leading to mitochondrial damage and increased generation of ROS [225]. A number of mutations that lead to familial PD have been linked to alterations in UPS function, including mutations in *SNCA* [226], *UCHL1*^227^, and *PRKN* [228]. In addition, *SNpc* samples from individuals with sporadic PD show decreased levels of alpha proteasome subunits [229, 230], decreased expression of the proteasome regulatory molecule PA700^222^, and reduced hydrolyzing activities of the 20/26S proteasome [223]. Experimental models have further implicated the UPS in PD pathophysiology, with iPSCs from PD patients and pesticide-induced animal models of PD-like degeneration showing impairment in UPS activity [231–233]. These findings have contributed to an understanding that aberrant protein aggregation in neurons directly promotes neurodegeneration. However, the UPS has also been shown to regulate immune function with the ability to both activate and dampen inflammasome activation [234]. This has led to a growing understanding that UPS dysfunction in immune cells may contribute to neurodegeneration in PD by driving inflammatory responses [235, 236].

Recent studies have demonstrated that inhibition of proteasome activity causes increased microglial activation in experimental models. KD of *HACE1*, which encodes an E3 ubiquitin-ligase involved in the UPS, was shown to exacerbate LPS-induced inflammation in BV2 microglia [237]. In addition, BV2 cells treated with siRNA for *HACE1* showed increased neurotoxicity during co-culture with SH-SY5Y cells compared to vehicle treated BV2 cells, indicating the neuroprotective effect of this protein [237]. Furthermore, KD of *HACE1* in MPTP-treated mice led to more severe motor deficits than MPTP treatment alone [237]. In line with these findings, administration of proteasome inhibitors in rat models of α-synuclein overexpression causes significant microgliosis and α-synuclein aggregation [238, 239]. Even in wild type rats, systemic exposure to proteasome inhibitors was sufficient to recapitulate parkinsonian motor features and DAN degeneration [240]. These findings provide strong evidence that disrupted protein homeostasis in microglia contributes to neuroinflammation and neurotoxicity.

Dysregulated protein homeostasis has also been reported in the peripheral blood of PD patients. Activity of the 20 S proteasome and expression of the ubiquitin conjugating enzyme E2 were found to be lower in PD PBMCs compared to controls [241]. Furthermore, lower 20 S proteasome activity in PBMCs was correlated with longer disease duration and greater severity of motor symptoms. No changes were reported with AD status [241], suggesting that this biomarker of metabolic dysfunction in PBMCs could be specific for PD and have clinical value for predicting disease progression. In addition, DJ-1 isoforms in whole blood from PD patients were shown to have a higher number of 4-hydroxy-2-nonenal post-translational modifications (PTMs) relative to AD patients or NHCs [242]. These PTMs are associated with inhibition of 20 S proteasome activity, and interestingly the frequency of 4-hydroxy-2-nonenal PTMs was positively correlated with PD disease severity [242]. Together, these results point towards systemic UPS defects in PD, and peripheral blood may serve as an accessible biofluid for evaluating PD-related defects in protein homeostasis.

## Conclusion

Cellular metabolic dysfunction represents a crucial component of the pathogenic cascade in PD. Mounting literature describes widespread disruption in energy homeostasis in immune cells, and these deficits converge on aberrant inflammation in both the central and peripheral immune systems. The emerging data suggest that metabolic reprogramming towards increased glycolytic activity is likely to be both a cause and a consequence of excessive inflammation in PD, setting the stage for a feed-forwards inflammatory milieu which renders DANs particularly vulnerable to degeneration. A number of genetic and environmental causes of PD converge on mitochondrial dysfunction, including deficits in ETC activity, ROS homeostasis, and mitochondrial respiration. In addition, changes in autophagic activity and lysosomal health have been shown to increase energetic demand. These facets of cellular metabolic dysfunction, many of which were first noted in neurons in PD, have now been shown to extend to immune cells in both the CNS and the periphery. Moreover, energy insufficiency appears to prime immune activation, leading to increased proinflammatory responses to primary insults. As growing attention is directed towards therapeutics targeting metabolism in PD, these strategies may be both directly neuroprotective as well as serve to mitigate the hostile inflammatory environment produced by activated immune cells.

We have highlighted here multiple studies demonstrating the success of pharmacologic interventions targeting glycolysis, mitophagy, and lysosomal pathways in preclinical PD models. However, considerable effort is still required to understand how these metabolic processes in immune cells interact and contribute to neurodegeneration. While significant attention has been directed towards metabolic changes in microglia, astrocytes remain a relatively understudied area with significant gaps in knowledge that must be addressed. Additionally, the field must devote future research towards assessing the feasibility and effectiveness of using immune-targeted therapies to rescue immunometabolic deficits and potentially delay or slow PD progression. Given the extensive parallels in metabolic dysfunction between the peripheral immune system and the central nervous system, the peripheral immune system may provide a non-invasive window into the brain for evaluating central bioenergetic deficits in PD. As we move beyond merely identifying bioenergetic deficits and begin to develop strategies to restore function, the field will need a cell-type-specific understanding of the complex pathways that converge on immunometabolic dysfunction in PD.

## Data Availability

Not applicable.
